# Differences between individuals with schizophrenia or obsessive-compulsive disorder and healthy controls in social cognition and mindfulness skills: A controlled study

**DOI:** 10.1371/journal.pone.0225608

**Published:** 2019-12-11

**Authors:** Yolanda López-del-Hoyo, Manuel González Panzano, Guillermo Lahera, Paola Herrera-Mercadal, Mayte Navarro-Gil, Daniel Campos, Luis Borao, Héctor Morillo, Javier García-Campayo

**Affiliations:** 1 Instituto de Investigación Sanitaria Aragón (IISAragon), Zaragoza, Spain; 2 University of Zaragoza, Zaragoza, Spain; 3 Primary Care Prevention and Health Promotion Research Network, RedIAPP, Madrid, Spain; 4 Faculty of Medicine, University of Alcalá, IRyCIS CIBERSAM, Madrid, Spain; 5 Universitat Jaume I, Castellón, Spain; 6 Miguel Servet University Hospital, Zaragoza, Spain; Univdersity Hospital of TübingenUniversitatsklinikum Tubingen, GERMANY

## Abstract

The study of social cognition (SC) has emerged as a key domain of mental health, supporting the notion that poorer performance in SC tasks is linked to psychopathology, although most studies have primarily addressed only schizophrenia (SZ). Some recent studies have also shown deficits of SC in obsessive-compulsive disorder (OCD) patients; however, little is known about how individuals with OCD may differ on SC performance from individuals with SZ. Moreover, initial research in this field suggests that mindfulness skills may be related to SC abilities such as theory of mind (ToM), emotion processing and empathy. Given the potential benefits of mindfulness for treating OCD and SZ, further efforts are needed to understand the association between mindfulness and SC in these populations. The main objective of this study was to compare samples of patients with SZ and OCD to healthy controls (HCs) on several social cognition (SC) domains and mindfulness measures. In total, 30 outpatients diagnosed with SZ, 31 outpatients diagnosed with OCD and 30 healthy controls were assessed in emotion recognition (the Eyes Test), ToM (the Hinting Task), attributional style (the Ambiguous Intentions and Hostility Questionnaire), empathy (the Interpersonal Reactivity Index) and dispositional mindfulness (the MAAS and the FFMQ). Both clinical groups showed poorer performance in emotion recognition and ToM than the HCs. The OCD and SZ patients did not significantly differ in impairment in SC, but the OCD group had higher scores in attributional style (intentionality and anger bias). With regard to mindfulness, the results found lower levels of acting with awareness for the HCs than for either clinical group and higher non-reactivity to inner experience for the HCs than for the individuals with OCD; the results also yielded significant correlations between SC and mindfulness. In conclusion, these findings revealed that SC abilities were impaired in the SZ and OCD groups compared to the HC group, suggesting a similar disrupted pattern in both clinical groups. Aspects of dispositional mindfulness were differentially associated with SC, which may suggest their potential role in novel transdiagnostic interventions.

## Introduction

The study of social cognition (SC) has drawn the attention of researchers, emerging as a key domain for the understanding and treatment of mental health problems [1,2]. SC can be understood independently from neurocognition (i.e., attention, language, and executive functions) and can be considered a mediator between neurocognition and social behaviour [3]. SC involves a set of mental operations underlying social interactions [4] and the capacity to understand oneself, others, social situations and interactions in a social world [5]. More particularly, it is the ability to perceive, process, interpret and generate responses to the intentions, dispositions and behaviours of others [4]. SC comprises four areas: emotion processing, social perception, theory of mind/mental state attribution (ToM), and attributional style/bias [6].

Evidence has supported the notion that poorer SC performance is linked to psychopathology, although most studies have primarily addressed only schizophrenia (SZ) [3,7,8]. Impairments in SC have been widely reported in individuals with SZ and have manifested as difficulties in identifying emotions, feeling connected to others, inferring people’s thoughts and reacting emotionally to others; these impairments are strong determinants of the degree of impaired daily functioning facing individuals with SZ [9]. Patients with SZ showed larger deficits across SC domains than healthy controls (HC) [1,10,11], with large effect sizes for ToM (*d* = -.96) and emotion recognition (*d* = .91) [1]. Recent studies have confirmed that the impairment or deterioration in ToM–the ability to infer the mental states of others, including intentions, dispositions and beliefs [12,13]–or mentalization plays a crucial role in the psychopathological process of SZ [3]. The literature has also reported impairments in the perception of social cues, emotion recognition and attributional style in individuals with SZ [3,9]. Empathy impairments have also been demonstrated in individuals with SZ, and these impairments have been viewed as an emergent phenomenon that depends on multiple components of SC [9,14–16]. Despite SC having clear implications in SZ and the fact that its impairment has been well documented, SC impairment may also play an important role in other mental disorders, as there is evidence suggesting that such deficits in SC may contribute to psychosocial deterioration and impact other cognitive abilities in many clinical conditions, thus appearing as a core cognitive phenotype [1,17].

Obsessive-compulsive disorder (OCD) is associated with significant disruptions in functioning across multiple settings (work, home and social life), leading to significant impairment in these important areas of daily living [18]. The World Health Organization ranks OCD as one of the 10 most handicapping conditions by lost income and decreased quality of life [19]. Since SC has been recognized as an important driver of functional outcomes in other conditions, it has also become an important study target in OCD research [17]. Furthermore, it has been suggested that deficits in SC may be common in patients with basal ganglia abnormalities, including those with Huntington’s disease, Parkinson’s disease and, hypothetically, OCD [20]. However, studies on SC in individuals with OCD are still relatively scarce. The results of a meta-analysis across different anxiety disorders showed deficits in emotion recognition (*d* = -.16), ToM (*d* = -.30) and attributional style (*d* = -.53) in individuals with OCD compared to HCs [17]. Whereas most of the studies assessed emotion recognition (N = 14), only two studies assessed ToM, and one study assessed attributional style; it was therefore concluded that such limited findings restricted the possibility of generalizing the performance of OCD patients across SC domains. One of those studies [21] found that OCD patients’ performances were worse in several ToM tasks than were those of HCs and revealed significant differences in “advanced” ToM abilities–which require third-order ToM skills (e.g., he knows that they think he will lie) and seem to be related to reduced memory capacity–with no differences found in “basic” ToM abilities assessed with first- and second-order ToM tasks. Contrary to those findings, a pilot exploratory study showed that OCD patients did not have lower social cognitive abilities but had dysfunctional metacognitive profiles – referred to as increased self-referential thinking with heightened attention to one’s own thinking processes and automatic thoughts – that may contribute to their psychosocial impairment via greater cognitive rigidity and poorer cognitive flexibility [22]. Recent findings support the notion that OCD is associated with ToM impairment and indicates significantly poorer ToM abilities in individuals with OCD than in HCs [23–25]. Interestingly, the ToM deficits observed in OCD patients could not be attributed to other neurocognitive dysfunctions, indicating that social and non-social cognition are distinct from each other empirically and neurobiologically, although they are related [23,26–28].

Difficulties in empathy have also been reported in OCD patients, particularly for negative emotional valence [29]. Compared to 130 HCs, OCD patients (N = 107) scored significantly lower on perspective taking and significantly higher on personal distress, which are specific facets of empathy [30]. OCD has also been linked to several cognitive dysfunctions [31].

Given the social impairment and social cognitive deficits seen in OCD patients, together with the implications of SC, further research is needed to explore SC abilities in this population and compare population to groups with other clinical conditions. Little is known about the differences in SC performance between individuals with OCD and individuals with SZ. A different SC profile is expected between the two populations based on different hypotheses about the mechanism underlying SC impairment. Therefore, one interesting research approach would be to investigate SC deficit in OCD patients and compare these patients with the individuals with SC impairments widely studied in the literature, e.g., SZ patients, where the SC profile has been widely documented.

As far as we know, there are no controlled studies comparing these two disorders that would allow the identification of specific SC profiles. This is an important field of research, given that SZ and OCD share clinical features and have high rates of comorbidity–OCD symptoms are commonly observed in individuals with SZ [32]. Whitton & Henry [33] found that the presence of comorbid obsessive-compulsive symptoms in individuals with a primary diagnosis of SZ was related to poorer performance in SC measures (emotion recognition and ToM), although these associations disappeared after controlling for more general SZ-psychopathology indices scores. Other authors have shown that patients with schizo-obsessive disorder did not differ in attention, executive functions or memory measures from those with SZ [34].

Recently, an exponential number of studies have found that mindfulness skills–the ability to pay attention, on purpose, in the present moment, non-judgementally [35]–are related to SC abilities such as ToM, emotion processing and empathy [36–39]. Thus, the way people pay attention to present-moment experiences affects the way they see and interact with the world [40]. Mindfulness-based interventions (MBIs) have shown potential benefits for the treatment of OCD and SZ [41–46]. Nevertheless, more research is required to identify the therapeutic components of mindfulness skills across diverse populations [45]. Based on a previous study conducted on a general population exploring relations between dispositional mindfulness and SC [47], we propose that mindfulness skills (i.e., the awareness of internal and external experiences by broadening one’s perspective without automatically reacting) will also be related to the ability to perceive, interpret and generate responses to the intentions, dispositions, emotions and behaviours of others (i.e., the core social cognition domains) in a clinical condition, particularly in SZ and OCD. Therefore, results confirming these assumptions would imply that mindfulness may be a useful technique for modifying how a person perceives the world by enhancing SC. This would be especially important for clinical populations in which SC abilities are impaired (i.e., SZ and OCD populations), which may further benefit from developing mindfulness skills through MBIs. Furthermore, identifying how specific facets of mindfulness are related to SC domains in the clinical population should foster a better comprehension of the social mind and behaviour in SZ and OCD patients and therefore contribute to enhancing SC performance.

Ultimately, the results of this study may shed light on the understanding of SC deficits and their potential association with dispositional mindfulness in SZ and OCD patients. In addition, exploring relationships between SC and mindfulness variables in different populations may provide insights for the implementation of specific training based on different mechanisms of action and intervention approaches.

The main objective of this study was to compare SC and OCD patients to healthy controls on several social cognition outcomes and mindfulness measures. Our hypothesis was that 1) both SZ and OCD patients will show poorer performance than the controls (healthy subjects) and that each group would show a specific profile –the extent to which SC is impaired or unaffected in each experimental group –which would reveal specific deficits in SC abilities in SZ and OCD patients; 2) the SZ group will show poorer performance than the OCD group on SC dimensions; and 3) the four areas of SC studied (i.e., emotion recognition, ToM, attributional/cognitive biases and empathy) will be affected in SZ patients, while ToM is expected to be unaffected in OCD patients–according to those studies suggesting affections on advanced ToM but not on basic ToM abilities [21]. A secondary objective was to explore the association between SC and dispositional mindfulness measures. We expected to find that the SC measures significantly correlated with dispositional mindfulness. The correlation between dispositional mindfulness and SC was expected to have a positive direction, with greater dispositional mindfulness correlated with greater performance in SC tasks or the inverse, where lower SC abilities are related to lower dispositional mindfulness levels.

## Material and methods

### Participants and procedure

The total sample comprised 91 participants (30 SZ patients; 31 OCD patients; 30 HCs). Patients diagnosed with SZ (N = 30) were recruited from several health facilities, such as the acute psychiatric care unit of the Miguel Servet University Hospital (Zaragoza, Spain) and the Nuestra Señora del Pilar Psychiatric Hospital (Zaragoza, Spain). Patients diagnosed with OCD (N = 31) were recruited from the Hospital Universitario de Bellvitge (HUB) (Barcelona, Spain). Both clinical samples were receiving the usual care for acute phases. The inclusion criterion for the clinical sample was a clinical diagnosis (i.e., OCD or SZ) according to the DSM-IV or ICD-10 by expert psychiatrists or clinical psychologists from the recruitment centres. The exclusion criteria for the clinical sample were visual disturbances that hindered carrying out the experimental tasks and positive symptomatology (i.e., visual or auditory hallucinations). SZ patients showing active phases of positive symptomatology were arranged according to times when positive symptomatology was not active. Neither duration of disease nor pharmacological treatment were exclusion criteria in this study. The HC group comprised healthy volunteers from the community with no psychiatric or neurological disorders who were able to read and understand Spanish with no visual disturbances that hindered the performance of the experimental tasks (N = 30). In relation to the experimental tasks, the researcher ensured that the participants did not fail in the different measures included in the study as a consequence of visual disturbance or misunderstandings by asking questions and monitoring their performance. Despite patients being assessed at different health facilities, the experimental procedure was conducted by a single researcher to avoid instructor bias and to ensure experimental consistency. Moreover, the experimental procedure was manualized and protocolized to guarantee that the researcher read the same instructions to all the participants.

### Instruments

*Emotion recognition* was measured by the revised Spanish version of the *Reading the Mind in the Eyes Test* (RMET, or Eyes Test), which is a measure comprising 36 photographs of the eye region of the faces of different actors and actresses. Respondents were asked to choose which of four words best described what the person in the photograph was thinking or feeling, and one point was assigned for each correct response, with scores ranging from 0 to 36 [12,48]. The Eyes Test, which assesses the ability to read subtle facial cues indicating the emotional state of another person, is highly recommend and has been used in previous studies [49]. For the sample in this study, the alpha value (α) was .961.

*ToM* was measured using the *Hinting Task* [50], which assesses the ability of respondents to infer the true intention behind indirect speech utterances throughout ten short passages reflecting an interaction between two characters [51]. The total score ranges from 0 to 20. A detailed description of the task, instructions and a correction form can be found in [51]. For the sample in this study, the α was .844

*Attributional style* was measured through the *Ambiguous Intentions and Hostility Questionnaire* (AIHQ) [52], which comprises 15 short vignettes reflecting negative outcomes that vary in intentionality (i.e., intentional, accidental and ambiguous intention). Respondents were asked to read each vignette, imagine the scenario happening to them and write down (i) the reason the other person or persons acted in a particular manner (Hostility Bias, AIHQ-HB subscale), (ii) whether the other person or persons performed the action on purpose (Intentionality Bias, AIHQ-IS subscale), (iii) how angry it made them (the respondents) feel (Anger Bias, AIHQ-AS subscale), (iv) how much they would blame the other person or persons (Blame Scale, AIHQ-BS subscale), and (v) how they would respond to the situation (Aggressivity Bias, AIHQ-AB subscale). For the sample in this study, the α ranged between .929 and .985 for the subscales (Hostility Bias, α = .929; Intentionality Bias, α = .895; Anger Bias, α = .918; Blame Scale, α = .908; Aggressivity Bias; α = .900).

*Empathy* was assessed using the *Interpersonal Reactivity Index* (IRI) [53–55], which comprises 28 items scored on a Likert scale ranging from 0 (“doesn’t describe me at all”) to 4 (“describes me very well”). The IRI assesses four components of empathy, including (i) fantasy (F) (i.e., the tendency to identify with fictitious characters), (ii) perspective taking (PT), (iii) empathic concern (EC), and (iv) personal distress (PD) in the face of others’ suffering. For the sample in this study, the α ranged between .505 and .622 for the subscales (fantasy, α = .576; perspective taking, α = .584; empathic concern, α = .622; personal distress, α = .505).

*Dispositional mindfulness* was measured through the *Mindful Attention Awareness Scale* (MAAS) and the *Five Facet Mindfulness Questionnaire* (FFMQ-SF). The MAAS is a 15-item scale that assesses an individual’s dispositional capacity to be aware and conscious during every moment of the day [56,57]. The alpha value for the sample in this study was .913. The FFMQ-SF [58] is a 24-item short-form version of the FFMQ [59–62] that assesses five different facets of mindfulness: (i) *observing*, which refers to an individual’s capacity to pay attention to internal and external experiences such as sensations, thoughts, and emotions; (ii) *describing*, which assesses the ability to describe events and personal responses in words; (iii) *acting with awareness*, which involves focusing on the activity being carried out instead of behaving automatically; (iv) *non-judging of inner experience*, which refers to the ability to take a non-evaluative stance towards thoughts and feelings; and (v) *non-reactivity to inner experience*, which involves allowing thoughts and feelings to come and go without getting caught up in or carried away by them [61]. The alpha values for the sample in this study were .898 for the total score and ranged between .699 and .728 for the subscales (observe, α = .728; describe, α = .724; awareness, α = .792; non-judging, α = .699; non-reactivity, α = .811).

*Depression and anxiety*. The *Hospital Anxiety and Depression Scale* (HADS) [63,64] is a self-report tool that contains 14 items measured on a 4-point Likert scale that assess anxiety and depression. The Spanish version of HADS has shown good psychometric properties for both psychiatric and healthy samples [65,66]. For the sample in this study, the alpha value was .833.

### Ethics

All research involving human participants was approved by The Clinical Research Ethics Committee of Aragon (Zaragoza, Spain), which also approved the study protocol. All clinical investigations were conducted according to the principles expressed in the Declaration of Helsinki, and subsequent modifications and the Madrid Declaration of the World Psychiatric Association. Participants gave written informed consent prior to their inclusion in this study to publish the results.

### Statistical analyses

Differences between groups in sociodemographic data were explored using chi-square (χ2) tests for categorical variables and analysis of variance (ANOVA) for continuous variables. Normality assumptions were explored using the Shapiro-Wilk test (for each experimental group, N < 50), the Kolmogorov-Smirnov (K-S) test (for the total sample, N > 50), skewness and kurtosis indices, and normality plots (Q-Q plots). Homoscedasticity was explored by Levene’s test and Box’s M test for equivalence of covariance matrices. Sensitivity analyses were conducted to ensure the adequacy and robustness of the statistical methods applied [37]. Parametric methods were reported since they could be considered robust and reliable due to the normal distribution of variables and classical assumptions being satisfied. Univariate analysis of covariance (ANCOVA) and multivariate analysis of covariance (MANCOVA), to control for significant differences in sociodemographic data, were performed to investigate the differences between groups. The effect sizes of the main effects were reported by partial eta squared (ηp.^2^). Statistically significant effects were followed up by multiple post hoc comparisons adjusted by the Dunn-Sidak method. These statistical tests have shown their robustness regardless of violations of the required assumptions when group sizes are equal [67]. Cohen’s *d* effect sizes and their 95% confidence intervals were calculated and reported for significant between-group comparisons [68–70]. Separate Pearson correlations were performed to explore the association between social cognition and dispositional mindfulness measures. Partial correlation analyses were also performed to control for sociodemographic variables (i.e., sex, and education). Significant correlations (*p* < .005) and those higher than *r* > .30 are reported. A Bonferroni approach to adjust the significance level of the Pearson correlation for multiple comparisons, in which the relationship between two variables is said to be significant when the p-value is less than .05 divided by the number of pairs of correlated variables, was performed. As we are examining correlations between 17 variables (i.e., 138 pairs), the corrected p-value is .0004. Data analyses were conducted using IBM SPSS Statistics 23.0 for Windows.

## Results

Table 1 shows the sociodemographic data of this study. Statistically significant between-group differences were found in all sociodemographic variables (all *p*s < .05), except for age.

**Table 1:** Sociodemographic characteristics of the sample.

	SZ n = 30	OCD n = 31	HC n = 30	Statistics
**Age**				F _(2,89)_ = 2.251
*Mean (SD)*	43.60 (10.82)	40.17 (11.95)	46.37 (11.22)	*p* = .111
**Sex, *n* (%)**				
*Male*	18 (60%)	20 (64.5%)	7 (23.3%)	*X*^2^_(6)_ = 12.337
*Female*	12 (40%)	11 (35.5%)	23 (76.7%)	*p* < .010
**Education, *n* (%)**				
*Uneducated*	3 (10%)	1 (3.2%)	0 (0.0%)	*X*^2^_(6)_ = 29.941
*Primary*	15 (50.0%)	7 (22.6%)	8 (26.7%)	*p* < .001
*Secondary*	11 (36.7%)	13 (41.9%)	3 (10.0%)	
*University*	1 (3.3%)	10 (32.3%)	19 (63.3%)	
**Marital status *n* (%)**				
*Single*	27 (90.0%)	16 (41.9%)	7 (23.3%)	*X*^2^_(6)_ = 29.609
*Married*	1 (3.3%)	13 (51.6%)	18 (60.0%)	*p* < .001
*Divorced*	2 (6.7%)	1 (3.2%)	4 (13.3%)	
*Widowed*	0 (0.0%)	1 (3.2%)	1 (3.3%)	
**Employment**				
*Unemployed*	10 (33.3%)	11 (32.5%)	2 (6.7%)	*X*^2^_(6)_ = 38.791
*Employed*	6 (20.0%)	9 (29.0%	26 (86.7%)	*p* < .001
*Retired*	0 (0.0%)	2 (6.5%)	2 (6.7%)	
*Disability*	14 (46.7%)	9 (29.0%)	0 (0.0%)	

Note. SD = Standard deviation. SZ = Schizophrenia group. OCD = Obsessive-compulsive disorder group. HC = Healthy control group.

The results showed statistically significant differences between the three groups for performance in the SC measures (Table 2). For empathy assessed by the IRI, significant differences were found between groups (F(8,156) = 4.565; *p* < .001; ηp.^2^ = .190), specifically on the EC and PD subscales. The HC group scored lower than the SZ group on EC (*p* < .05) and the OCD group on PD (*p* < .001) (Table 2). Sex was a significant covariate for the EC subscale (F(4,77) = 3.778; *p* < .01; ηp.^2^ = .164). When comparing performance in the Eyes Test on emotion recognition, there were significant differences between groups (F(2,84) = 73.651; *p* < .001; ηp.^2^ = .637), where the score was significantly higher for the HC group than for the SZ and OCD groups (all *p*s < .001). Statistically significant differences were also found for the Hinting Task assessing ToM (F (2,84) = 10.623; *p* < .001; ηp.^2^ = .202), with higher scores in the HC group than in the SZ (*p* < .001) and OCD (*p* < .05) groups. For attributional style in the AIHQ, the analysis yielded significant differences between groups (F (10,162) = 3.405; *p* < .001; ηp.^2^ = .174), particularly on the IS and AS subscales, where the OCD group scored significantly lower than the SZ group (*p* < .10).

**Table 2:** Comparisons between patients with schizophrenia or obsessive-compulsive disorder and healthy controls on social cognition outcomes and mindfulness-related measures.

	SZ n = 30	OCD n = 31	HC n = 30	Statistics	Significant comparisons	Cohen’s *d* [95% CI]
**IRI**						
*IRI_PT*	23.18 (5.87)	22.93 (5.44)	26.55 (4.69)	F = 2.630		
*IRI_FS*	21.21 (4.42)	22.77 (5.61)	21.62 (5.15)	F = .696		
*IRI_EC*	23.82 (4.38)	26.93 (4.23)	28.52 (3.37)	F = 3.76***^a^	HC > SZ*	.63 [.11, 1.14]
*IRI_PD*	21.07 (5.94)	22.13 (5.85)	16.59 (4.46)	F = 8.710***	HC < OCD***	-1.05 [-1.58, -.51]
**Eyes Test**	13.10 (6.67)	9.87 (4.06)	26.63 (4.68)	F = 73.651***	HC > OCD*** HC > SZ***	3.78 [2.94, 4.62] 2. 32 [1.66, 2.97]
**Hinting Task**	11.50 (4.77)	14.23 (4.26)	17.27 (2.69)	F = 10.623***	HC > OCD* HC > SZ***	.84 [.32, 1.36] 1.47 [.90, 2.04]
**AIHQ**						
*HB*	13.77 (8.19)	17.10 (5.78)	17.50 (8.69)	F = 1.536		
*IS*	40.63 (18.11)	51.48 (14.82)	40.83 (7.43)	F = 6.226**	OCD > SZ**	.65 [.13, 1.16]
*AS*	39.10 (17.55)	46.71 (14.49)	39.13 (8.12)	F = 3.538*	OCD > SZ**	.47 [-.04, .98]
*BS*	40.33 (17.75)	44.90 (14.76)	38.63 (6.52)	F = 1.498		
*AB*	17.70 (9.33)	18.03 (6.36)	21.70 (8.36)	F = 3.195		
**MAAS**	3.51 (1.53)	3.77 (1.10)	3.77 (.92)	F = .314		
**FFMQ**						
*Observing*	12.39 (3.93)	12.25 (3.38)	14.63 (3.09)	F = 2.674		
*Describing*	16.00 (4.43)	15.82 (4.39)	16.83 (4.04)	F = .759 ^d^		
*Awareness*	17.83 (4.26)	17.11 (4.95)	14.50 (3.68)	F = 5.998**^c^	HC < OCD* HC < SZ**	-.59 [-1.12, -.07] -.83 [-1.40, -.27]
*Non-judgement*	11.13 (3.93)	10.04 (3.66)	11.47 (3.82)	F = 1.655^b^^c^		
*Non-reactivity*	16.04 (4.31)	12.14 (4.58)	16.60 (2.76)	F = 11.241**	OCD < HC*** OCD < SZ**	-1.17 [-1.73, -.62] -.86 [-1.44, -.29]
**HADS**						
*Anxiety*	7.40 (5.41)	11.90 (5.26)	5.97 (2.83)	F = 14.497***^d^	OCD > HC*** OCD > SZ***	1.40 [.82, 1.94] .83 [.31, 1.36]
*Depression*						
*Total*	14.03 (9.25)	19.55 (8.76)	8.20 (4.39)	F = 14.030***^d^	OCD > HC*** OCD > SZ**	1.61 [1.03, 2.19] .61 [.09, 1.12]

Note. SD, Standard deviation; SZ, Schizophrenia group; OCD, Obsessive-compulsive disorder group; HC, Healthy control group; 95% CI, Confidence interval; IRI, Interpersonal Reactivity Index; FS, Fantasy; PT, Perspective-taking; EC, Empathic concern; PD, Personal distress; AIHQ, Ambiguous Intentions and Hostility Questionnaire; HB, Hostility Bias; IS, Intentionality Bias; AS, Anger Bias; BS, Blame Scale; AB, Aggressivity Bias; MAAS, Mindful Attention Awareness Scale; FFMQ, Five Facet Mindfulness Questionnaire. HADS, Hospital Anxiety and Depression Scale.

*p < .05

**p < .01

*** p < .001.

^a^ Sex was a significant covariate.

^b^ Marital status was a significant covariate.

^c^ Employment was a significant covariate.

^d^ Educational level was a significant covariate.

With regard to comparisons between mindfulness measures, no significant differences were found between the three groups on the MAAS total score (F(2,84) = .314; *p* = .732; ηp.^2^ = .007). For the FFMQ, the analysis showed statistically significant differences between groups (F(10,142) = 4.356; *p <* .001; ηp.^2^ = .235). The HC group scored lower on the awareness facet than the OCD (*p* < .05) and SZ (*p* < .01) groups. Employment was a significant covariate for the awareness measure (F(1,77) = 10.701; *p* < .01; ηp.^2^ = .126). Moreover, the OCD group reported lower scores for the non-reactivity measure than the HC (*p* < .001) and SZ (*p* < .01) groups, where employment (F(1,74) = 4.734; *p* < .05; ηp.^2^ = .60) and marital status (F(1,74) = 5.241; *p* < .05; ηp.^2^ = .66) were significant covariates.

Correlation analysis for the total sample showed a significant association between the SC measures and dispositional mindfulness. The MAAS was significantly correlated with the subscales from the AIHQ (all *p*s < .01) [(HB: r = .300), (IS: r = .401), (AS: r = .308), (BS: r = .349) and (AB: r = .356)]. For the FFMQ, significant correlations were found between the observing measure and the Eyes Test (r = .322; *p* < .01) and the AIHQ [(IS: r = .400; p < .01), (AS: r = .311; *p* < .01), (AB: r = .333; p < .01)]. The describing facet was negatively correlated with the PD subscale from the IRI (r = -.389; *p* < .01). Significant correlations were found for the non-reactivity facet and the Eyes Test (r = .363; *p* < .05) and for the AB subscale from the AIHQ (AB: r = .402; *p* < .01).

Partial correlation controlling differences in sociodemographic variables (i.e., sex and education) showed significant associations for the non-reactivity facet from the FFMQ with the PD subscale (r = -.332; *p* < .01) and the IS subscale from the AIHQ (r = -.313; *p* < .01). The non-judgement facet from the FFMQ was significantly correlated with several subscales from the AIHQ [(IS: r = -.341; p < .01), (AS: r = -.342; *p* < .01), (BS: r = -.320; p < .01)]. Table 3 shows correlations between SC and dispositional mindfulness for each group (HC, OCD and SZ groups). The results from the correlation analyses were not significant after Bonferroni correction (all *p*s > .0004).

**Table 3:** Correlations between social cognition and dispositional mindfulness measures for each group.

**HC**						
	*MAAS*	*Observing*	*Describing*	*Awareness*	*Non-judgement*	*Non-reactivity*
*IRI PT*	-.009	.141	.290	.265	.252	.184
*IRI FS*	-.240	.123	.127	.188	.226	-.029
*IRI EC*	-.170	.060	.029	-.065	.150	-.394*
*IRI PD*	-.230	.277	-.059	.106	.360	-.092
*Eyes test*	.022	.137	.316	.213	.125	-.216
*Hinting Task*	-.090	-.121	-.409*	.000	-.056	.108
*AIHQ HB*	-.204	.097	.036	.159	.177	.224
*AIHQ IS*	.157	.023	-.072	.313	.011	-.126
*AIHQ AS*	.028	-.140	.024	.081	.026	-.332
*AIHQ BS*	.150	-.241	.104	.126	.020	-.259
*AIHQ AB*	-.139	-.162	-.223	.003	.143	.181
**OCD**						
	*MAAS*	*Observing*	*Describing*	*Awareness*	*Non-judgement*	*Non-reactivity*
*IRI PT*	.103	-.208	-.021	.054	.010	.021
*IRI FS*	.202	.231	.139	-.037	-.203	-.019
*IRI EC*	-.229	-.206	.020	-.007	-.305	-.192
*IRI PD*	-.190	-.192	-.520**	-.380	-.146	-.114
*Eyes test*	-.186	-.096	-.151	-.291	.038	-.112
*Hinting Task*	.479**	.278	.203	.131	.354	.375*
*AIHQ HB*	.413*	.322	-.255	-.130	-.150	.276
*AIHQ IS*	.407*	.625**	.038	.072	-.140	.141
*AIHQ AS*	.217	.380*	-.248	-.232	-.241	-.063
*AIHQ BS*	.207	.377*	-.014	-.169	-.195	.059
*AIHQ AB*	.280	.424*	-.442*	-.321	-.178	.153
**SZ**						
	*MAAS*	*Observing*	*Describing*	*Awareness*	*Non-judgement*	*Non-reactivity*
*IRI PT*	.097	-.138	-.289	-.006	.064	.167
*IRI FS*	-.042	.059	-.020	-.284	-.533**	.051
*IRI EC*	.236	.055	-.194	.208	-.035	.110
*IRI PD*	-1.92	-.039	-.452*	-.649**	-.431*	-.092
*Eyes test*	.291	.244	.196	-.038	-.061	.357
*Hinting Task*	.236	.133	-.071	.338	.265	-.030
*AIHQ HB*	.549**	.193	-.054	-.330	-.290	.385*
*AIHQ IS*	.483**	.511**	-.229	-.467*	-.430*	.484**
*AIHQ AS*	.444*	.497**	-.265	-.359	-.416*	.620**
*AIHQ BS*	.498**	.470**	-.151	-.460*	-.440*	.598**
*AIHQ AB*	.672**	.454*	.170	.225	.058	.581**

Note. Significant correlations are set in bold. IRI, Interpersonal Reactivity Index; FS, Fantasy; PT, Perspective-taking; EC, Empathic concern; PD, Personal distress; AIHQ, Ambiguous Intentions and Hostility Questionnaire; HB, Hostility Bias; IS, Intentionality Bias; AS, Anger Bias; BS, Blame Scale; AB, Aggressivity Bias; MAAS, Mindful Attention Awareness Scale; FFMQ, Five Facet Mindfulness Questionnaire. HADS, Hospital Anxiety and Depression Scale

*p < .05.

**p < .01. The corrected p-value for Bonferroni correction is < .0004.

## Discussion

The main objective of this study was to compare SZ patients, OCD patients and HCs on SC and mindfulness measures. As expected, significant differences were found between groups for the SC measures. Overall, both clinical groups showed poorer performance than the HC group, revealing significant differences in emotion recognition and ToM. These findings are in line with previous research that has pointed out SC impairments in individuals with OCD and SZ, which to date have always been studied separately from healthy subjects [1,11,17]. For the empathy measure, which, according to a number of authors, reflects increased motivation to help others in a selfless attempt to increase well-being [71,72], significantly higher empathy scores were obtained from the HC group than from the SZ group, specifically for empathic concern (the other-focused emotion of caring for others who are suffering) [53]. Lower personal distress scores were obtained from the HC group than from the OCD group, a finding that was also in line with those of previous studies [30]. With regard to the influence of sociodemographic variables on SC, our data indicate that sex was a significant covariate for emphatic concern, which is congruent with the findings of studies pointing out the role of sex on empathy skills [73,74].

When comparing the SZ and OCD patients on SC measures, the results showed significant differences only for attributional style (intentionality bias and anger bias), with higher scores in the OCD group. These findings are consistent with those of studies suggesting cognitive (i.e., interpretative) biases in OCD patients [17,75,76]. Moreover, our data indicated that the OCD and SZ patients did not differ in the impairment observed in other relevant SC domains, such as emotion recognition and ToM, contrary to studies suggesting differences in ToM, which would be hypothetically unaffected in individuals with OCD [21]. Therefore, these findings may suggest a similar altered pattern in these skills that supports and explains the evidence of shared clinical features and high rates of comorbidity between OCD and SZ [32,34], which conflicts with the hypothesis that individuals with SZ will show poorer SC abilities than individuals with OCD. These findings also support the proposal that SC domains should be considered potential clinical markers that may serve to establish effective transdiagnostic interventions that address the general mechanisms of action rather than specific diagnoses [1,77].

With regard to mindfulness outcomes, significant differences were found in a number of mindfulness facets. A suggestive finding was that the HC group had lower levels of *acting with awareness* than both clinical groups and higher levels of *non-reactivity to inner experience* than the OCD group. On the one hand, it is important to consider that there is evidence suggesting a link between maladaptive forms of awareness and psychopathology [78–80] that may presumably be related to paying attention to the present moment in a judgemental manner. On the other hand, *non-reactivity to inner experience* has been shown to be a key feature because it implies a greater perceptual distance from internal and external cues, which in turn leads to a decentring perspective of experience along with associated reductions in emotional reactivity [38,81–84]. These data agree with previous research that has highlighted the role of the non-reactivity facet on SC domains [85]. Nevertheless, more research is needed to truly comprehend these relationships. Regarding the influence of sociodemographic factors, our results showed that employment was a significant covariate for awareness and non-reactivity, while marital status was significant for non-reactivity. These findings, together with those studies exploring the effect of sociodemographic factors on mindfulness practices [86], might support the necessity to take sociodemographic characteristics into account in mindfulness research.

With regard to our secondary objective, as expected, our study showed an association between SC and dispositional mindfulness measures, indicating that greater values of dispositional mindfulness are associated with better performance in SC tasks and vice versa. Specifically, dispositional mindfulness (MAAS total score) was correlated with attributional style/bias, whereas several mindfulness facets were differentially correlated with each of the SC measures. Thus, *observing* (i.e., sensitivity to internal and external experiences, sensations, thoughts and emotions) was related to emotion recognition and attributional style/bias. The *describing* facet was inversely associated with personal distress (one of the empathy subscales), revealing that a greater ability to describe events and personal responses in words seemed to be related to experiencing lower personal distress when confronting the suffering of others. *Non-reactivity to inner experience* was correlated with emotion recognition and attributional style/bias, more specifically with aggressivity bias. These results indicate that SC abilities and dispositional mindfulness are linked in various ways that may imply different mechanisms of action and therefore different approaches for training in each specific domain. For example, based on these data, it could be suggested that greater emotion recognition is related to increased abilities for observing and not reacting to internal and external cues, whereas aggressivity bias is more associated with non-reactivity skills. Moreover, personal distress when facing suffering from others, a component of empathy, would be further decreased by means of training and describing skills from a mindfulness perspective. Therefore, these distinct skills can be acquired using different meditation practices that have demonstrated differential effects [83].

Furthermore, consideration should be given to partial correlation analyses that reveal changes in the associations between dispositional mindfulness and SC when controlling for differences in sociodemographic variables (i.e., sex and education), which may imply a role of sociodemographic factors and their importance to better understanding the research on SC and mindfulness. Specifically, these results showed significant inverse correlations for non-reactivity to inner experiences with the personal distress scale (empathy subscale) and the intentionality bias scale, indicating that a greater ability to avoid reacting to inner experience is associated with lower personal distress when facing the suffering of others; lower intentionality bias scores indicate a greater ability to determine whether another person or other persons performed an action on purpose. Moreover, a greater ability for non-judging inner experiences was associated with lower intentionality bias, lower anger bias (or how angry the situation made them feel), and lower intention to blame others. According to the Bishop et al. [87] definition of a two-component model for mindfulness – (1) attention focused to the present moment (2) in a particular way – the facets of non-reactivity and non-judgement are the attitudinal components of adopting a particular orientation towards one’s experiences in the present moment, which is an orientation characterized by curiosity, openness and acceptance. Our findings may highlight the influence of dealing with inner experiences with non-reactivity and non-judgement to develop greater SC skills in the general population and in SZ and OCD populations. Therefore, interventions with a more in-depth focus on how to manage internal experiences with a “mindful” attitude, beyond their attentional benefits, may impact how we perceive and navigate the social world by enhancing SC in terms of perceiving, interpreting and generating responses to the intentions, dispositions, emotions and behaviours of others (i.e., the core SC domains), particularly for clinical populations in which deficits in SC play a central role, such as SZ and OCD populations.

Dispositional mindfulness and SC abilities were related differently in each sample. One of the most interesting findings involved the differences on the attributional scale, where significant correlations were found only in the clinical samples, which may suggest a role of attributional biases and their relation to the ability to deal with present-moment experiences with openness and acceptance in daily life, particularly for OCD and SZ patients, owing to its influence on social functioning [11]. Furthermore, attributional bias scales were mainly related to the attentional component of mindfulness (observing facet) in the OCD sample, while both attentional (observing and awareness) and attitudinal (non-judgement and non-reactivity) facets were associated with attributional biases in the SZ sample. These results may reveal differences in how OCD and SZ patients process social information and how their clinical features affect deficits in daily social life functioning. Another relevant issue is that performance on the ToM task was associated with the overall dispositional mindfulness skill in the OCD sample, suggesting that mindfulness is further related to the ability to infer the mental states of others, including intentions, dispositions and beliefs, and the OCD patients showed greater overall dispositional mindfulness that the SZ patients. However, our data cannot be generalized, and correlational analyses do not allow inferential causes, although they may inform us about different SC profiles and their relationships with mindfulness skills.

In summary, the findings of this study have important implications. First, our results revealed that patients with SZ or OCD had significant impairments in SC abilities that may explain their social deficits. In addition, both clinical populations showed a similar altered pattern in these skills, which supports and explains the evidence of shared clinical features and high rates of comorbidity between OCD and SZ. Second, in consideration of these findings, transdiagnostic interventions focused on SC domains may be useful across a range of clinical conditions. A greater understanding of SC deficits may therefore provide opportunities to develop effective transdiagnostic interventions [1,9]. Third, the observed correlations between SC and dispositional mindfulness may point to the potential role of including mindfulness components in the transdiagnostic interventions comprehensively targeting SC abilities, specifically those focused on how to deal with inner experiences in an adaptative mode (i.e., non-reacting and non-judging the experiences), which seems to help in perceiving, interpreting and responding to social interactions where SC is key. In this regard, several studies have shown the efficacy of MBIs for the treatment of OCD and psychosis patients [88–91], which may support the hypothesis by suggesting additional benefits of including both SC and mindfulness skills in OCD and SZ interventions. However, more studies ensuring robustness and quality – i.e., randomized controlled trials –are needed to investigate these issues.

There are some limitations that should be mentioned. First, given that this study was a cross-sectional design (i.e., comparing samples at one time point), causal inferences are not possible. Second, a full neuropsychological evaluation was not performed; therefore, there was no exploration of the role of neuropsychological features and other cognitive variables (i.e., working memory or verbal fluency) as trait markers and possible endophenotypes of these disorders, which would have been in line with studies that have noted the relevance of attentional and executive functions in OCD and SZ patients [36,92]. In this regard, future studies are needed to further explore SC abilities in relation to other cognitive functions for several clinical disorders, as authors have claimed their association (e.g., correlations between ToM and language abilities) [93]. Third, although SC was assessed (i.e., emotion recognition, ToM, and attributional style) and an empathy measurement was also included in this study, there was no specific instrument to assess the social perception domain. Fourth, the scores for each particular emotion in the emotion recognition task were not individually analysed. This is an interesting issue based on studies indicating that emotion regulation may differ in individuals with OCD depending on emotional valence [29,30]. Given that the experimental groups were not homogeneous and that this may have affected the results, it is also important to consider significant differences in sociodemographic data (i.e., gender, education, marital status, employment) in the studied sample as a limitation. Although sociodemographic data were introduced as covariables and partial correlations were performed to control and study their possible effect in the principal comparisons, further efforts are needed in future studies to match the groups for the given sociodemographic factors, for example, recent evidence regarding the impact of gender on SC [94]. Moreover, some specific clinical data of the participants (e.g., the duration of disease or pharmacological treatments) were not provided and could not be taken into account because the data were not accessible to the research team, which restricts the replicability of the study. Potential moderators or mediators that may explain the results were not analysed (e.g., duration of illness, influence of depressive symptoms, comparing subgroups of SZ and OCD patients or exploring the impact of obsessive thought vs obsessive behaviour on SC). Further studies should address and explore significant predictors and mediators of SC abilities, which may contribute to the development of targeted interventions [95]. Finally, it is also important to mention that the results from the correlation analyses were not significant after Bonferroni adjustment (corrected *p-*value < .0004). However, multiplicity adjustments in exploratory trials may not be required because any positive findings from exploratory trials should undergo additional testing before changing clinical practice (100).

## Conclusions

Patients diagnosed with SZ and OCD showed significant impairments in SC abilities compared to healthy controls, indicating a similar altered pattern for both clinical groups. SC abilities and dispositional mindfulness are linked in different ways, which may imply different mechanisms of action and approaches to training in each domain. Specifically, transdiagnostic interventions, including both SC and mindfulness skills, may be a useful approach for the treatment of SZ and OCD, especially given that recent findings support the notion that awareness of internal and external experiences without automatically reacting and judging promotes greater ability to perceive, interpret and generate responses to the intentions, dispositions, emotions and behaviours of others (i.e., the core SC domains). These patients may further benefit from these kinds of interventions due to the SC deficits shown. Finally, our findings suggest the importance of dealing with inner experiences with non-reactivity and non-judgement to develop greater SC skills in the general population and in patients diagnosed with SZ and OCD.

## Supporting information

S1 File Experimental data. (SAV)
